# Case Report: Chemotherapy-free treatment with camrelizumab and anlotinib for elderly patients with *KRAS* and *TP53* mutated advanced lung cancer

**DOI:** 10.3389/fphar.2023.1026135

**Published:** 2023-01-12

**Authors:** Wenbo Qi, Dayong Xi, Yuping Bai, Le Liu, Yanling Ma, Zhenyu Yin, Hao Chen

**Affiliations:** ^1^ The Second Clinical Medical College of Lanzhou University, Lanzhou, China; ^2^ Department of Oncology Surgery, Lanzhou University Second Hospital, Lanzhou, China; ^3^ Key Laboratory of the Digestive System Tumors of Gansu Province, Lanzhou University Second Hospital, Lanzhou, China

**Keywords:** NSCLC, camrelizumab, anlotinib, *KRAS*, *TP53*, chemotherapy-free, case report

## Abstract

**Background:** Lung cancer is a major public health issue and an enormous burden on society in China. Most lung cancers occur in elderly patients with non-small cell lung cancer (NSCLC), and many factors limit their treatment options. Chemotherapy-free therapy can avoid psychological fear, treatment pain, and adverse reactions caused by chemotherapy. Patients with non-small cell lung cancer with tumour protein p53 (*TP53*) gene mutations or Kirsten rat sarcoma viral oncogene homologue (*KRAS*) gene mutations tend to be more sensitive to anlotinib or programmed cell death protein 1 (PD-1) drugs. However, Kirsten rat sarcoma viral oncogene homologue is a proto-oncogene downstream of the epidermal growth factor receptor (*EGFR*) gene; therefore, if the Kirsten rat sarcoma viral oncogene homologue gene has an activating mutation, EGFR-targeted drug resistance may occur. Further studies are needed to explore whether patients with dual Kirsten rat sarcoma viral oncogene homologue and tumour protein p53 mutations can be treated with targeted immunotherapy without chemotherapy.

**Case presentation:** A 74-year-old man was referred to the Lanzhou University Second Hospital due to chest tightness, shortness of breath, and weight loss for 2 months and was diagnosed with moderately to poorly differentiated adenocarcinoma. Laboratory examinations showed increased alpha-fetoprotein (AFP), carcinoembryonic antigen (CEA), cancer antigen (CA)-125, and CA199 levels, and gene sequencing indicated mutations in Kirsten rat sarcoma viral oncogene homologue and tumour protein p53. Immunohistochemical analysis showed positive PD-L1 and PD-1 expression. Peripheral blood immune checkpoint test using flow cytometry indicated that the PD-1 + CD8 levels were positive. After multi-disciplinary treatment, therapy with a combination of anlotinib and camrelizumab was initiated. Camrelizumab 200 mg was administered intravenously once every 3 weeks. Anlotinib 12 mg was administered orally daily before breakfast for 2 weeks with a week of rest in every cycle of 21 days. A reduction in alpha-fetoprotein, carcinoembryonic antigen, CA125, CA199, and CA724 levels was observed up to the first cycle, which decreased within the normal limits up to the second cycle and continued until the eighteenth cycle. The patient’s chest tightness, shortness of breath, weight loss, and other symptoms significantly improved following treatment. Computed tomography imaging showed that the neoplastic lesion was dramatically reduced. The patient is currently being followed-up for more than 2 years to evaluate the duration of the response.

**Conclusion:** Chemotherapy-free immunotherapy combined with targeted therapy is an effective treatment for advanced non-small cell lung cancer in elderly patients with Kirsten rat sarcoma viral oncogene homologue and tumour protein p53 mutations. Such therapies should be supported with further clinical studies with larger sample sizes.

## Introduction

Lung cancer ranks second in incidence and first in mortality worldwide (Sung et al., 2021), with non-small cell lung cancer (NSCLC) comprising 85% of all lung cancer cases (Govindan et al., 2006). Surgery, chemoradiotherapy, chemotherapy, immunotherapy, and targeted drugs are common treatment strategies (Gridelli et al., 2015). Elderly patients make up a large proportion of patients with lung cancer; their age, physical condition, and possible postoperative complications can significantly limit treatment options. A comprehensive assessment of the overall situation of patients aged ≥70 years using effective tools is required. It is worth mentioning that chemotherapy-free status can eliminate psychological fear, treatment pain, and chemotherapy-related adverse reactions in patients.

In cancer progression, the PD-L1/PD-1 axis plays an important role in the immune evasion mechanisms of cancer cells by regulating T cell activity (Pardoll, 2012). Immune checkpoint inhibitors (ICI) targeting PD-1, including nivolumab (Borghaei et al., 2015), pembrolizumab (Herbst et al., 2016), and camrelizumab (Jiangsu Hengrui Pharmaceuticals Co., Ltd.) (Zhou et al., 2021), have become effective treatment options for patients with NSCLC (Borghaei et al., 2015; Herbst et al., 2016; Zhou et al., 2021). Nivolumab significantly improved overall survival (OS) in patients with advanced NSCLC in two phase III trials (median OS, nivolumab vs. docetaxel: 12.2 months vs. 9.4 months) (Borghaei et al., 2015; Brahmer et al., 2015). Pembrolizumab also prolonged OS in the aforementioned patients, with at least 1% PD-L1 expression in tumour cells (median OS, pembrolizumab vs. docetaxel: 12.7 months vs. 8.5 months) (Herbst et al., 2016). Apatinib combined with camrelizumab demonstrated potent antitumour activity and acceptable toxicity in patients with advanced NSCLC as a second-line treatment (OS, 15.5 months; 95% CI, 10.9–24.5) (Zhou et al., 2021). Moreover, studies have shown that ICI efficacy in patients with Kirsten rat sarcoma viral oncogene homologue (*KRAS*) mutant NSCLC is similar to that in patients with other NSCLCs. PD-L1 expression is more predictive of ICI efficacy in patients with *KRAS*-mutant NSCLC than in those with other NSCLC (Jeanson et al., 2019). Immunotherapy has shown promising results for patients with NSCLC. However, the population that can benefit from this treatment is small. Hence, selecting the dominant population for specific immunotherapy is a matter of utmost importance.

Anlotinib (Focus V^®^ Jiangsu Chia Tai-Tianqing Pharmaceutical Co., Ltd.) is an oral small-molecule inhibitor of multiple receptor tyrosine kinases that has a broad inhibitory effect on tumour angiogenesis and growth (Sun et al., 2016). Anlotinib prolongs OS and is well-tolerated in Chinese patients with advanced NSCLC treated with third-line therapy (median OS, anlotinib vs. placebo: 9.6 vs. 6.3 months) (Han et al., 2018). Furthermore, patients with advanced NSCLC and tumour protein p53 (*TP53*) mutations treated with anlotinib showed longer progression-free survival (Fang et al., 2020). *TP53* mutations may be a biomarker for the selection of anlotinib therapy.

*KRAS* mutations are found in approximately 10%–15% of cases of NSCLC in Asia (Dearden et al., 2013; Yoshizawa et al., 2013). *KRAS* is the most frequently activated oncogene in NSCLC, and *KRAS*-mutant lung cancers have generally been associated with poorer OS than *KRAS* wild-type tumours (Mascaux et al., 2005; Johnson et al., 2013; Marabese et al., 2015; Jordan et al., 2017). Mutations in the tumour suppressor gene *TP53* are also common in lung cancer and are often accompanied by *KRAS* mutation (Ding et al., 2008; Pao and Girard, 2011; Gibbons et al., 2014). *TP53* and *KRAS* genes were found to have significant effects on PD-L1 expression, infiltration of immune T cells, and enhancement of tumour immunogenicity (Dong et al., 2017). The clinical efficacy of PD-1 inhibitors is significant in patients with *TP53* or *KRAS* mutations and *TP53*/*KRAS* mutations (Dong et al., 2017). In patients with *TP53*/*KRAS* co-mutation, the response rate of patients to anti-PD-1 is up to 57% (median progression-free survival (PFS), *TP53* and *KRAS* vs. *TP53* vs. *KRAS* vs. wild-type: 30 vs. 14.5 vs. 14.7 vs. 3.5 months) (Dong et al., 2017). *TP53* and *KRAS* mutations in patients with NSCLC may be effective predictors of immunotherapy efficacy.

Here, we report an elderly patient with *KRAS* and *TP53*-mutated advanced NSCLC adenocarcinoma for whom camrelizumab and anlotinib therapy was administered.

## Case presentation

**FIGURE 1:**
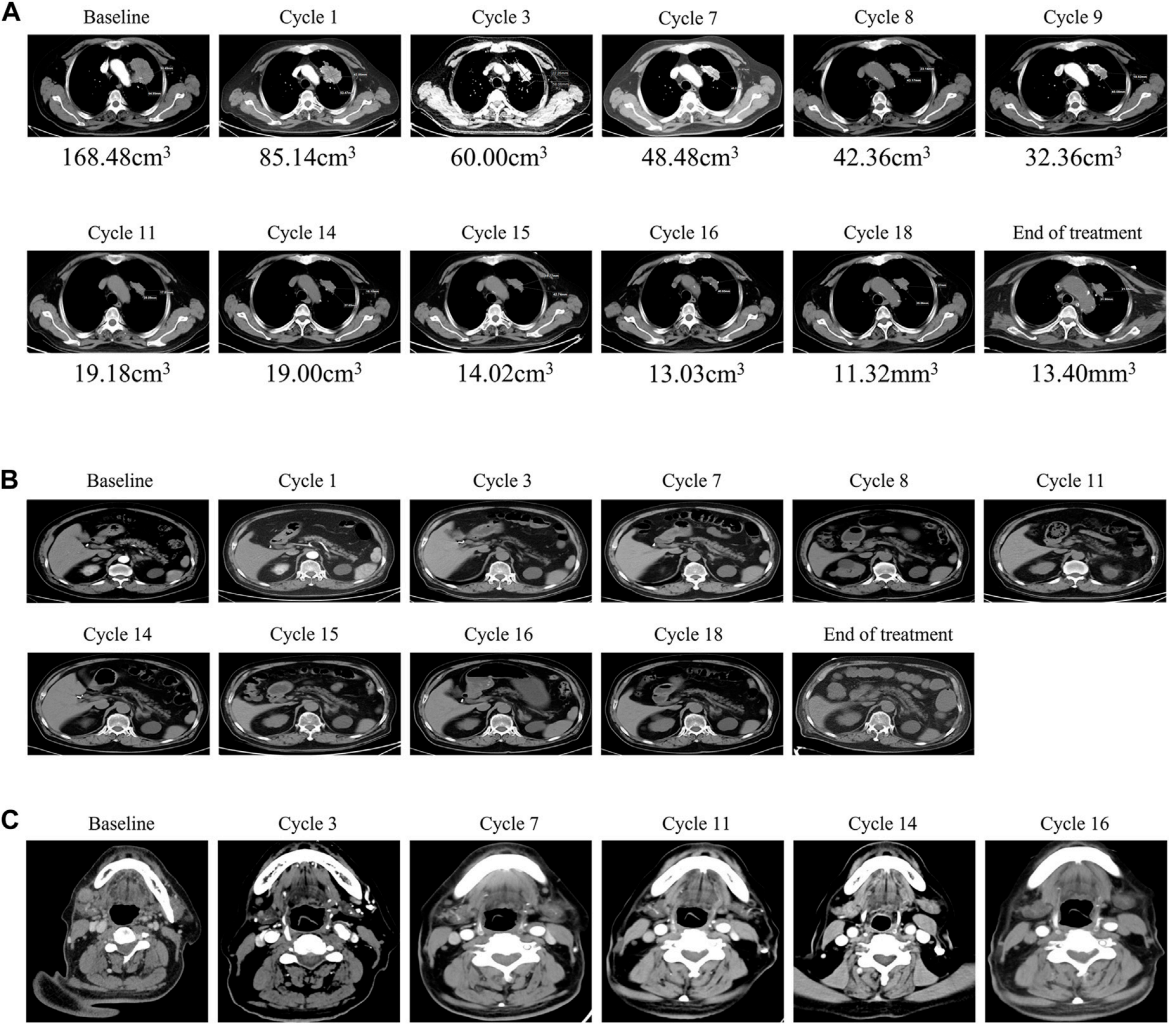
Changes in lung tumor and metastatic tumor during camrelizumab and anlotinib treatment. **(A)** CT showed that the lung tumor began to shrink after first cycle of treatment until the end of treatment. **(B)** CT showed that the volume of bilateral adrenal invasion lesions was significantly reduced after first cycle of treatment and disappeared after that. **(C)** CT showed that the volume of the right neck invasion lymph node lesion was significantly reduced to normal after three cycles until the end of treatment.

**FIGURE 2:**
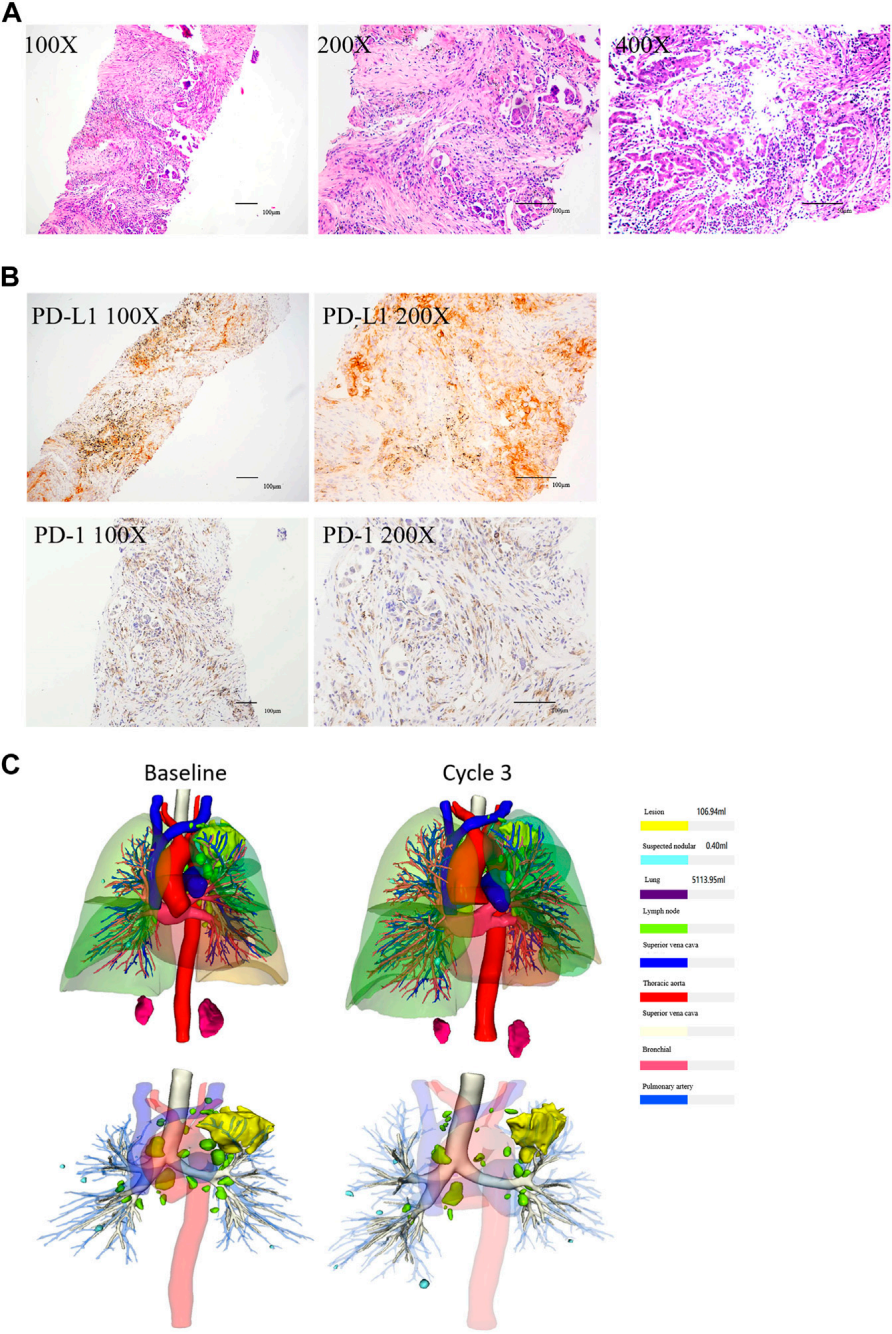
HE and IHC of NSCLC tissues, and the three-dimensional reconstruction of tumor. **(A)** HE showed that the tissues were moderately and poorly differentiated adenocarcinoma (From left to right: ×100 magnification, ×200 magnification, and ×400 magnification). **(B)** IHC showed that the expression of PD-L1 and PD-1 were positive (Left: ×100 magnification, right: ×200 magnification). **(C)** Three-dimensional reconstruction showed that the tumor shrinked after the third cycle.

On 28 April 2020, a 74-year-old man was referred to our hospital with complaints of chest tightness, shortness of breath, and weight loss for 2 months. Computed tomography (CT) (Figure 1A) revealed a mass in the left lung with bilateral adrenal invasion (Figure 1B) and right neck lymph node invasion (Figure 1C). AFP (8.95 ng/ml; normal value < 7.00), CEA (15.03 ng/ml; normal value < 3.40), CA125 (175.10 U/ml; normal value < 35.00), CA199 (79.27 U/ml; normal value < 27.00), and CA724 (6.03 U/ml; normal value < 6.90) levels were abnormal (Supplementary Figure S1). The patient underwent endobronchial ultrasonography-guided transbronchial needle aspiration, and the tumour was diagnosed as a moderately to poorly differentiated adenocarcinoma (90% for acinar adenocarcinoma; 10% for solid adenocarcinoma; T4N3M1 stage IV) (Figure 2A) with mutations in *KRAS* and *TP53* (Table 1). The former mutation was a missense mutation in exon two of the gene, which is predicted to have led to the functional activation of its encoded protein, whereas the *TP53* mutation was a splicing mutation in the intron of this gene, which was predicted to have caused the inactivation of protein function. Results of the immunohistochemical analysis showed that the PD-L1 expression score (SP263) in tumour cells was 0.8% and PD-1 staining (Servicebio: GB12338) of CD8 cells in tumor lesions was positive (Figure 2B). The results of peripheral blood immune checkpoint tests demonstrated that the proportions of LAG3+CD8, TIM3+CD8, PD-1+CD8, and CD3-CD19-CD14+CD16-HLA-DR levels were higher than the reference range (Supplementary Table S1).

**TABLE 1:** Gene mutations associated with tumor targeted therapy.

Gene	Base changes	Amino acid change	Function prediction	Mutations in abundance (%)
*KRAS*	Missense mutation in exon 2: c.57G>C	p.L19F	The activation	8.19
*TP53*	Splicing mutations in introns: c.376-1G>A	—	The deactivation	6.84

Since the patient was elderly with poor constitution, and his family members refused chemotherapy, and given that the patient had driver gene *TP53* and *KRAS* mutations, it was recommended through multi-disciplinary discussion that immunotherapy combined with targeted therapy should be administered. Consequently, the patient was treated with a combination of camrelizumab and anlotinib. Camrelizumab (200 mg) was administered intravenously once every 3 weeks. Anlotinib (12 mg) was administered orally every day before breakfast for 2 weeks with a week’s rest in every cycle of 21 days. The patient provided written informed consent for treatment.

**FIGURE 3:**
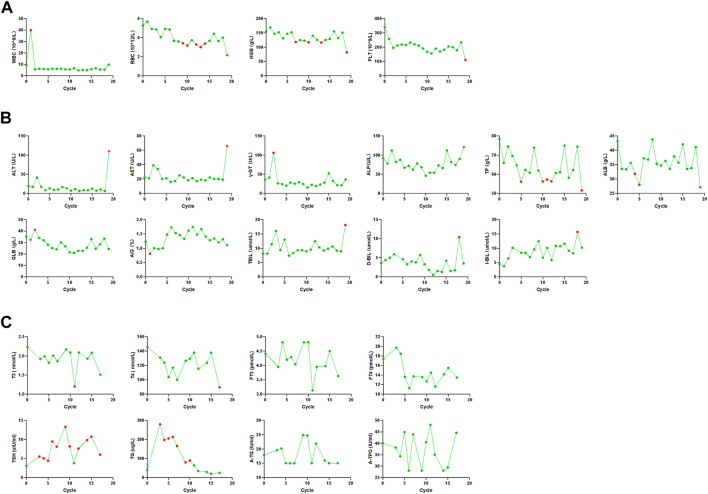
Changes in blood cell counts, liver function, and thyroid function. **(A)** Blood cell counts was almost within normal limits until the end of treatment. **(B)** Liver function was almost within normal limits until the end of treatment. **(C)** Thyroid function was almost within normal limits until the end of treatment.

After one cycle of combination therapy, AFP, CEA, CA125, CA199, and CA724 serum levels decreased to 6.22, 10.17 ng/ml, 148.20, 42.84 U/ml, and 1.26 ng/ml, respectively (Supplementary Figure S1). Three-phase multidetector CT highlighted a marked dimensional reduction of infiltrating focality in the left lung (from 168.48 mm^3^ to 85.14 mm^3^) (Figure 1A) and adrenal glands (Figure 1B). Furthermore, the patient’s chest tightness, shortness of breath, weight loss, and other symptoms significantly improved. To evaluate the efficacy more clearly, we used a three-dimensional reconstruction system (Hubei Inlook Technology Co., Ltd.) to precisely measure the volume of the lesion and found that the tumour size significantly decreased after treatment (Figure 2C). To evaluate safety, changes in blood cell counts (Supplementary Table S2; Figure 3A), liver function (Supplementary Table S3; Figure 3B), and thyroid function (Supplementary Table S4; Figure 3C) were detected and are shown. Changes in pancreatic function are shown in Figure 2.

Treatment continued up to 18 cycles (till 26 November 2021). CT demonstrated a good response with the reduction of mass in the lung (Figure 1A), adrenal (Figure 1B), and right neck lymph node invasion (Figure 1C). The tumour marker values remained within the normal range (Supplementary Figure S1). Three-dimensional reconstruction revealed a marked reduction in infiltrating focality in the left lung (Figure 2C). Blood cell counts (Supplementary Table S2; Figure 3A), liver function (Supplementary Table S3; Figure 3B), thyroid function (Supplementary Table S4; Figure 3C), and pancreatic function (Supplementary Figure S1) were nearly within normal limits.

**FIGURE 4:**
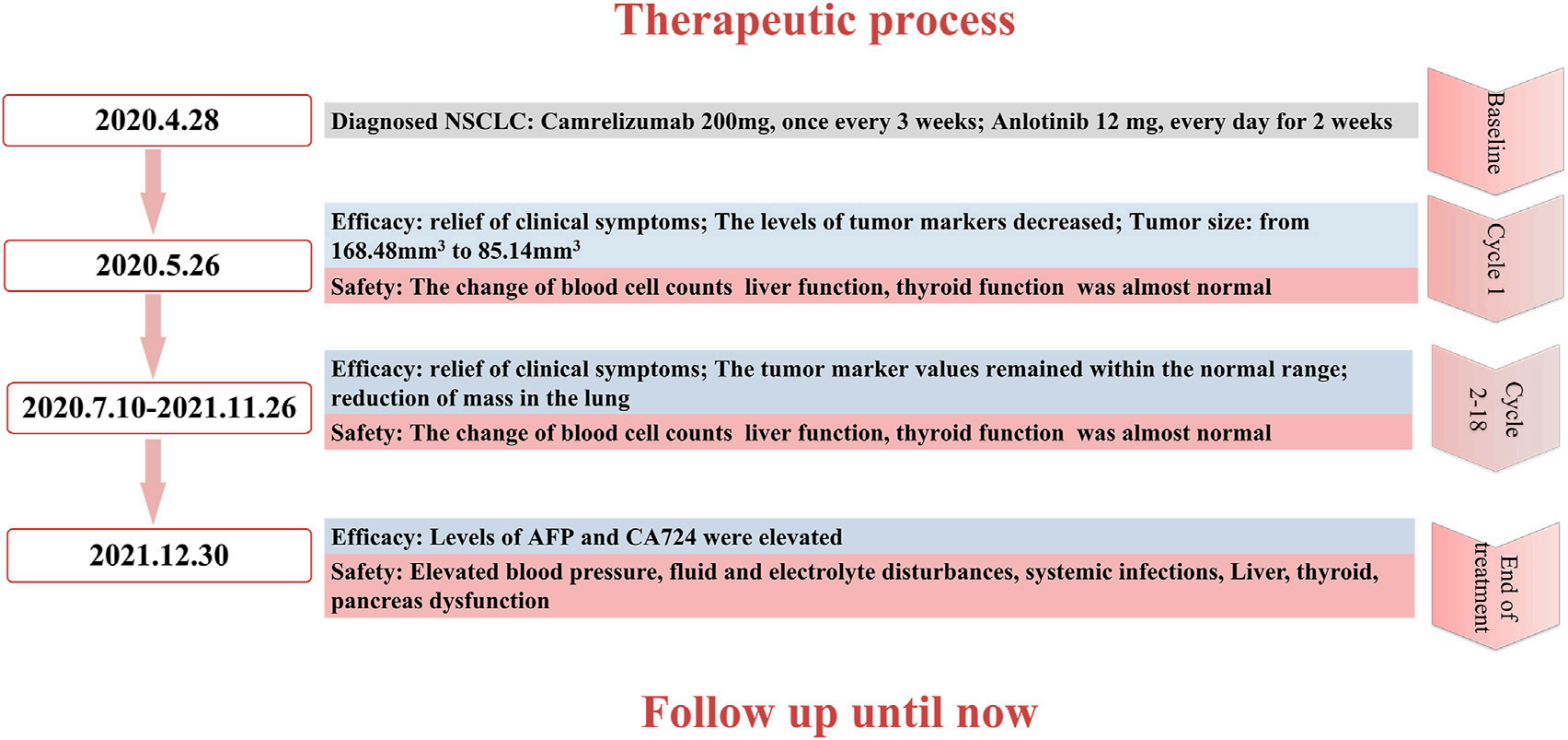
The whole therapeutic process including treatment strategy, efficacy and side effects.

After cycle 19 (2021.12.30), the patient did not visit the hospital on time due to personal reasons. Furthermore, AFP and CA724 levels were elevated. Blood cell counts (Supplementary Table S2; Figure 3A), liver function (Supplementary Table S3; Figure 3B), and thyroid function (Supplementary Table S4; Figure 3C) were not within normal limits. The patient developed elevated blood pressure, fluid and electrolyte disturbances, systemic infections, and liver, thyroid, and pancreatic dysfunctions. This condition is mainly due to the side effects of drugs. Treatment ended owing to subjective and objective reasons. The patient is still being followed-up. The therapeutic process is shown in Figure 4.

During the treatment, the patient had good compliance, no serious adverse reactions, and the minor adverse reactions were acceptable, manageable, and improved after symptomatic treatment. The study was reviewed and approved by the Institutional Ethics Committee of the Lanzhou University Second Hospital.

## Discussion

The main first-line treatment for advanced NSCLC is chemotherapy, which mainly includes paclitaxel and gemcitabine. This case achieved a good treatment outcome without chemotherapy, indicating that chemotherapy is not necessary for some patients. The latest research showed that the objective response rate, disease control rate, median PFS, and 12-month OS rate of sindilizumab combined with anlotinib as a first-line therapy were 72.7%, 100%, 15 months, and 95.5% (Chu et al., 2021), respectively, effectively improving the survival time and life quality of first-line lung cancer patients and truly achieving a “chemotherapy-free” status. The results of this study are consistent with those of ours. However, individual cases may not be appropriate for the general patient.

Generally, the toxicity associated with treatment increases with age (Movsas et al., 1999; Atagi et al., 2012; Dawe et al., 2016). However, age is not the only limitation that excludes patients from the most effective treatment; other factors such as comorbidities, social and personal circumstances, and performance need to be collectively assessed (Ng et al., 2005). Simultaneously, clinical trials are being conducted to evaluate older patients (≥75 years of age) with stage III NSCLC, including frail, vulnerable, and healthy older patients, with quality-adjusted survival as the primary endpoint (Driessen et al., 2018). Considering the complex physical condition of elderly patients, multi-disciplinary evaluation is strongly recommended for all elderly NSCLCs patients. Chemotherapy-free treatment can reduce the physical burden of treatment, prolong survival, and the improve quality of life in elderly patients; thus, it is recommended for elderly patients with poor tolerance.

PD-1/PD-L1 antibodies significantly improved the rate of durable response. It also prolongs long-term survival with advanced NSCLC (Xia et al., 2019), with limited adverse effects. However, the overall objective response rate of second-line therapy was less than 20%, and the progression-free survival (PFS) was similar or worse than that of conventional second-line chemotherapy (Borghaei et al., 2015; Herbst et al., 2016; Rittmeyer et al., 2017). Patients who are not expected to benefit from monotherapy with PD-1/PD-L1 antibodies, such as patients with PD-L1 negative tumors or those refractory to first-line therapy, should be treated in combination. Recent studies have shown that anlotinib can change the tumor immune microenvironment by down-regulating the expression of PD-L1 on vascular endothelial cells (Liu et al., 2020). Antiangiogenic drugs can enhance tumor immune response, whereas immune checkpoint inhibitors can normalize blood vessels in the tumor microenvironment (Vanneman and Dranoff, 2012; Ciciola et al., 2020). Furthermore, preclinical studies have shown that the combination of immunotherapy and anti-angiogenic drugs can reprogram the tumour microenvironment and enhance anti-tumour efficacy by inhibiting tumour angiogenesis, promoting vascular normalisation and increasing tumour T lymphocytes infiltration (Manegold et al., 2017). Recent studies have shown that the results of sintilimab in combination with anlotinib as a first-line treatment for advanced NSCLC showed that the objective response rate was 72.7%, and the disease control rate was 100% (Chu et al., 2021). Anti-angiogenic drugs combined with immunotherapy have a synergistic effect in the treatment of various cancers, including advanced NSCLC (Manegold et al., 2017). In this case, we analyzed the efficacy and safety of anlotinib combined with camrelizumab (a PD-1 inhibitor) in the treatment of advanced NSCLC patients, and explored the synergistic effect of antiangiogenic agents and immunotherapy, both of which showed favorable results.

However, some patients may develop adaptive resistance to current immunotherapy regimen and many patients do not respond to this treatment. Combined with relevant references and Supplementary Table S1, the anti-LAG-3 antibody or an antibody cocktail (anti-LAG-3, anti-TIM-3 and anti-PD-1) may improve clinical outcomes to overcome immune resistance (Baumeister et al., 2016). Studies on patients with NSCLC have found that Treg cells expressing LAG-3 produce high levels of immunoregulatory cytokines IL-10 and TGF-β, and inhibit the activation of TIL (Camisaschi et al., 2010). LAG-3 binds to its ligand to form an immune checkpoint pathway independent of PD-1/PD-L1, leading to T cell dysfunction and tumor cell evasion of immune surveillance (Camisaschi et al., 2010). Preclinical cancer treatment models using LAG-3 blocking show that antigen-specific T cells at the tumor site are activated, tumor growth is inhibited, and tumor parenchyma is destroyed (Grosso et al., 2007). However, some studies have shown that although mice lacking LAG-3 alone will not produce spontaneous autoimmunity, mice lacking both LAG-3 and PD-1 will produce fatal systemic autoimmunity (Okazaki et al., 2011; Woo et al., 2012), which highlights the synergy of these two approaches in controlling T cell tolerance. T cells in tumor and chronic viral infection usually coexpress PD-1 and LAG-3, and the therapeutic effect of blocking PD-1 and LAG-3 jointly in tumor models is greater than that of blocking alone (Blackburn et al., 2009; Matsuzaki et al., 2010; Woo et al., 2012). Currently, clinical trials related to the treatment of NSCLC with LAG-3 antibody are ongoing [IBI110, IMP321]. TIM-3 is a promising inhibitory receptor among many emerging immune checkpoints. TIM-3 is expressed on CD4+ TH1 helper T cells and CD8+ Tc1 cytotoxic T cells, and is associated with the prognosis of various cancers, including NSCLC (Das et al., 2017). It has been reported that up-regulation of TIM-3 in NSCLC patients is one of the mechanisms of adaptive resistance to PD-1 inhibitor (Koyama et al., 2016). Preclinical studies in mouse tumor models have shown that anti-TIM-3 monotherapy can shrink tumors, and its combination with anti-PD-1 or anti-PD-L1 significantly reduces tumor load and improves anti-tumor immune response (Zhu et al., 2005; Ngiow et al., 2011). Several NSCLC drugs targeting TIM-3 (Cobolimab, INCAGN-02390, RO7121661, BGB-A425) are currently in clinical trials as monotherapy or in combination with anti-PD-1/PD-L1 drugs.

Why do only a few people benefit from targeted therapy? Among the factors used for assessment, biological markers may be the most important. The present case report describes a patient with advanced NSCLC involving a *KRAS* and *TP53* mutant, treated with camrelizumab and anlotinib. Gene detection and evaluation have extensively supported the efficacy of immunotherapy and targeted therapy in the absence of chemotherapy. Activation of *KRAS* mutations is reported to be considered as a driver of tumour progression. *KRAS* has been reported to be associated with increases in tumour-infiltrating lymphocytes, PD-L1 expression, and tumor mutational burden (Lee et al., 2018). There is evidence that *KRAS* mutation is a genetic marker of the benefit of emerging direct inhibitors and is combined with immunotherapy during clinical development (Adderley et al., 2019). *TP53* mutations are associated with genomic stability and defects in DNA damage repair (Soussi and Beroud, 2001). One study illustrated a significant response to anlotinib in patients with advanced NSCLC with *TP53* mutations (Fang et al., 2020). Dong et al. (2017) found that the *TP53*/*KRAS* co-mutation led to an increased PD-L1+/CD8A + ratio and longer PFS with PD-1 inhibitors (NCT01295827) (Cortez MA et al., 2015; Ji et al., 2016). In our case, PD-L1 expression was also positive and the proportions of LAG3+CD8, TIM3+CD8, PD-1+CD8, and CD3-CD19-CD14+CD16-HLA-DR levels were higher than the reference range. The patient in our case achieved encouraging efficacy with mutations in *TP53* and *KRAS*, further proving that these two genes can be used as indicators for immunotherapy and targeted therapy.

Unlike side effects such as rash, elevated blood pressure, liver dysfunction, and diarrhoea caused by targeted therapy, immunotherapy can produce immune-related side effects, such as fluid and electrolyte disturbances, immune pneumonia, immune hepatitis, systemic infections, and liver, thyroid, and pancreas dysfunction (Borghaei et al., 2015; Brahmer et al., 2015; Herbst et al., 2016; Rittmeyer et al., 2017). Furthermore, targeted therapy may cause tumours to develop drug resistance, and long-term application may reduce its efficacy. During treatment, the adverse reactions caused by these two drugs were tolerable. After 19 cycles, the patient did not attend the hospital on time for personal reasons and developed a series of adverse reactions, and the treatment was, therefore, ended. Generally, combining immunisation and targeted therapy significantly prolongs patient survival with a manageable safety profile.

To the best of our knowledge, there are few studies on PD-1 antibodies combined with targeted agents in patients with first-line NSCLC who did not receive chemotherapy. In view of its encouraging efficacy, durability, and safety profile, camrelizumab plus anlotinib represents a novel chemotherapy-free regimen for elderly patients with *TP53* and *KRAS* mutations.

## Data Availability

The original contributions presented in the study are included in the article/Supplementary Material, further inquiries can be directed to the corresponding author.

## Supplementary material

SUPPLEMENTARY FIGURE S1 A change of tumor markers and amylase level. The levels of AFP, CEA, CA125, CA199 and CA724 decreased to normal levels after the second treatment cycle and continued to the 19th cycle; The levels of AMY and AMY-U decreased to normal levels after the second treatment cycle until cycle 15.
