# Supplementary figures and images for: Case Report: Chemotherapy-free treatment with camrelizumab and anlotinib for elderly patients with KRAS and TP53 mutated advanced lung cancer
Source: Front Pharmacol. 2023 Jan 12;14:1026135. doi: 10.3389/fphar.2023.1026135 (PMC9878280; doi:10.3389/fphar.2023.1026135)

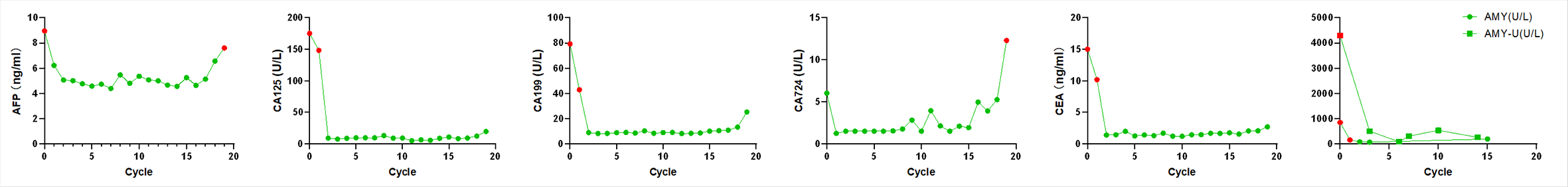

Supplement: Supplementary file 1 [file Image1.TIF]
