# Supplementary material for: Case Report: Chemotherapy-free treatment with camrelizumab and anlotinib for elderly patients with KRAS and TP53 mutated advanced lung cancer
Source: Front Pharmacol. 2023 Jan 12;14:1026135. doi: 10.3389/fphar.2023.1026135 (PMC9878280; doi:10.3389/fphar.2023.1026135)
Supplement: Supplementary file 2 [file DataSheet1.docx]

**Supplementary table 1** Immune checkpoint expression detection report.

| **Combination of tests** | **Number of positive cells (Per 10,000 cells)** | **Results** | **Reference range** |
| --- | --- | --- | --- |
| **CTLA4+CD8** | 0 | negative | <10 |
| **LAG3+CD8** | 43 | positive | <10 |
| **TIM3+CD8** | 25 | positive | <10 |
| **PD1+CD8** | 28 | positive | <20 |
| **CD3-CD19-CD14+CD16-HLA-DR** | / | positive | <19.38% |

**Supplementary table 2** Changes in blood cell counts.

| **Item** | **C0** | **C1** | **C2** | **C3** | **C4** | **C5** | **C6** | **C7** | **C8** | **C9** | **C10** | **C11** | **C12** | **C13** | **C14** | **C15** | **C16** | **C17** | **C18** | **C19** |
| --- | --- | --- | --- | --- | --- | --- | --- | --- | --- | --- | --- | --- | --- | --- | --- | --- | --- | --- | --- | --- |
| **WBC** | 9.22 | 39.90 | 5.59 | 6.14 | 5.95 | 5.59 | 6.14 | 5.95 | 6.10 | 5.57 | 5.50 | 6.53 | 4.86 | 5.15 | 4.93 | 5.79 | 6.54 | 5.48 | 5.41 | 9.74 |
| **GRAN%** | 0.65 | 0.92 | 0.42 | 0.46 | 0.48 | 0.42 | 0.46 | 0.47 | 0.57 | 0.54 | 0.51 | 0.58 | 0.52 | 0.57 | 0.59 | 0.60 | 0.59 | 0.64 | 0.57 | 0.70 |
| **LYM%** | 0.21 | 0.05 | 0.44 | 0.38 | 0.33 | 0.44 | 0.38 | 0.34 | 0.32 | 0.35 | 0.36 | 0.31 | 0.37 | 0.32 | 0.34 | 0.30 | 0.32 | 0.29 | 0.33 | 0.13 |
| **MONO%** | 0.09 | 0.03 | 0.09 | 0.07 | 0.07 | 0.09 | 0.07 | 0.08 | 0.08 | 0.07 | 0.08 | 0.07 | 0.08 | 0.07 | 0.06 | 0.07 | 0.08 | 0.06 | 0.07 | 0.13 |
| **EO%** | 0.05 | 0.00 | 0.03 | 0.09 | 0.11 | 0.03 | 0.09 | 0.11 | 0.03 | 0.03 | 0.04 | 0.04 | 0.04 | 0.03 | 0.02 | 0.03 | 0.02 | 0.02 | 0.02 | 0.03 |
| **BASO%** | 0.01 | 0.00 | 0.01 | 0.01 | 0.01 | 0.01 | 0.01 | 0.01 | 0.01 | 0.01 | 0.01 | 0.00 | 0.00 | 0.00 | 0.00 | 0.00 | 0.00 | 0.00 | 0.00 | 0.01 |
| **GRAN** | 5.96 | 39.66 | 2.37 | 2.81 | 3.17 | 2.37 | 2.81 | 2.78 | 3.47 | 3.02 | 2.81 | 3.76 | 2.51 | 2.95 | 2.89 | 3.47 | 3.83 | 3.48 | 3.10 | 6.81 |
| **LYM** | 1.95 | 2.04 | 2.48 | 2.30 | 2.13 | 2.48 | 2.30 | 2.02 | 1.92 | 1.95 | 2.00 | 2.02 | 1.79 | 1.65 | 1.66 | 1.75 | 2.06 | 1.56 | 1.77 | 1.25 |
| **MONO** | 0.81 | 1.33 | 0.52 | 0.44 | 0.48 | 0.52 | 0.44 | 0.45 | 0.48 | 0.39 | 0.45 | 0.45 | 0.37 | 0.38 | 0.29 | 0.40 | 0.52 | 0.32 | 0.40 | 1.31 |
| **EO** | 0.44 | 0.03 | 0.17 | 0.54 | 0.73 | 0.17 | 0.54 | 0.67 | 0.20 | 0.16 | 0.21 | 0.28 | 0.17 | 0.15 | 0.08 | 0.15 | 0.11 | 0.10 | 0.12 | 0.32 |
| **BASO** | 0.06 | 0.14 | 0.05 | 0.05 | 0.05 | 0.05 | 005 | 0.03 | 0.03 | 0.05 | 0.03 | 0.02 | 0.02 | 0.02 | 0.01 | 0.02 | 0.02 | 0.02 | 0.02 | 0.05 |
| **RBC** | 5.27 | 5.68 | 4.93 | 4.86 | 4.06 | 4.93 | 4.86 | 3.67 | 3.60 | 3.41 | 3.17 | 3.73 | 3.29 | 3.00 | 3.38 | 3.67 | 4.42 | 3.64 | 4.01 | 2.16 |
| **HGB** | 155 | 169 | 147 | 152 | 131 | 147 | 152 | 118 | 125 | 123 | 117 | 140 | 126 | 116 | 125 | 129 | 155 | 132 | 151 | 82 |
| **PLT** | 339 | 258 | 196 | 212 | 218 | 215 | 233 | 220 | 211 | 191 | 167 | 156 | 190 | 170 | 182 | 205 | 200 | 179 | 234 | 111 |

**WBC: white blood cell count, GRAN%: neutrophil ratio, LYM%: lymphocyte ratio, MONO%: monocyte ratio, EO%: eosinophil ratio, BASO%: basophils ratio, GRAN: neutrophil, LYM: lymphocyte, MONO: monocyte, EO: eosinophil, BASO: basophils, RBC: red blood cell, HGB: hemoglobin, PLT：platelet**

**Supplementary table 3** Changes in liver function

| **Item** | **C0** | **C1** | **C2** | **C3** | **C4** | **C5** | **C6** | **C7** | **C8** | **C9** | **C10** | **C11** | **C12** | **C13** | **C14** | **C15** | **C16** | **C17** | **C18** | **C19** |
| --- | --- | --- | --- | --- | --- | --- | --- | --- | --- | --- | --- | --- | --- | --- | --- | --- | --- | --- | --- | --- |
| **ALT** | 19 | 17 | 41 | 17 | 8 | <15 | 9 | 10 | 16 | 13 | 7 | 11 | 6 | 8 | 8 | 12 | 7 | 10 | 6 | 110 |
| **AST** | 22 | 21 | 39 | 34 | 20 | 21 | 16 | 17 | 25 | 22 | 18 | 21 | 17 | 19 | 18 | 22 | 20 | 20 | 19 | 66 |
| **γ-GT** | 36 | 41 | 106 | 26 | 24 | 20 | 28 | 25 | 29 | 24 | 15 | 22 | 19 | 23 | 28 | 52 | 32 | 21 | 21 | 36 |
| **ALP** | 92 | 78 | 112 | 82 | 88 | 67 | 72 | 62 | 78 | 68 | 46 | 54 | 54 | 73 | 66 | 112 | 82 | 74 | 90 | 121 |
| **TP** | 78.4 | 65.9 | 74.5 | 69.6 | 64.7 | 56.1 | 62.3 | 60.8 | 73.9 | 61.9 | 56.2 | 57.2 | 56.2 | 60.6 | 61.1 | 75.0 | 58.1 | 62.2 | 74.4 | 51.5 |
| **ALB** | 43.2 | 33.5 | 33.4 | 35.6 | 31.8 | 28.0 | 37.2 | 36.8 | 43.8 | 35.3 | 34.7 | 36.3 | 33.5 | 37.9 | 35.7 | 42.1 | 33.5 | 33.8 | 41.1 | 27.1 |
| **GLB** | 35.2 | 32.4 | 41.1 | 34.0 | 31.9 | 28.1 | 25.1 | 24.0 | 30.1 | 26.6 | 21.5 | 20.9 | 22.7 | 22.7 | 25.4 | 32.9 | 24.6 | 28.4 | 33.3 | 24.4 |
| **A/G** | 1.23 | 0.81 | 1.00 | 0.97 | 1.00 | 1.48 | 1.73 | 1.53 | 1.46 | 1.33 | 1.61 | 1.74 | 1.48 | 1.67 | 1.41 | 1.28 | 1.34 | 1.21 | 1.31 | 1.11 |
| **TBIL** | 8.1 | 8.1 | 11.4 | 16.00 | 9.4 | 13.0 | 7.4 | 8.3 | 9.3 | 9.3 | 8.9 | 9.5 | 12.4 | 10.3 | 9.2 | 9.8 | 10.6 | 9.2 | 8.9 | 18.1 |
| **D-BIL** | 3.6 | 4.4 | 5.0 | 5.9 |  | 4.6 | 3.3 | 4.0 | 3.8 | 5.7 | 3.3 | 1.8 | 0.5 | 1.5 | 1.3 | 4.2 | 1.5 | 1.7 | 10.4 | 3.5 |
| **I-BIL** | 4.5 | 3.7 | 6.4 | 10.1 |  | 8.4 | 8.4 | 6.9 | 9.6 | 12.4 | 6.7 | 10.1 | 5.8 | 10.8 | 10.7 | 11.6 | 9.1 | 8.2 | 15.7 | 10.2 |
| **CHOL** | 3.42 | 4.64 | 3.83 | 5.83 | 4.79 | 5.24 | 5.11 | 4.77 | 6.49 | 4.32 | 4.48 | 4.88 | 4.77 | 5.77 | 6.04 | 6.90 | 4.94 | 6.13 | 7.02 | 1.39 |

**ALT: alanine aminotransferase，AST:** [**aspartate aminotransferase**](javascript:;)**，γ-GT: Gamma-glutamyl transferase，ALP: alkaline phosphatase，TP: total protein， ALB:** [**albumin**](javascript:;)**， GLB: globulin， TBIL: total bilirubin，D-BIL：direct bilirubin， I-BIL** **indirect bilirubin，CHOL：total cholesterol**

**Supplementary table 4** Changes in thyroid function

| **Item** | **C0** | **C3** | **C4** | **C5** | **C6** | **C7** | **C9** | **C10** | **C11** | **C12** | **C14** | **C15** | **C17** |
| --- | --- | --- | --- | --- | --- | --- | --- | --- | --- | --- | --- | --- | --- |
| **T3** | 2.23 | 1.92 | 1.99 | 1.82 | 2.01 | 1.86 | 2.17 | 2.09 | 1.20 | 2.09 | 1.93 | 2.08 | 1.51 |
| **T4** | 145.40 | 130.80 | 123.80 | 103.70 | 116.80 | 100.10 | 126.50 | 129.40 | 137.90 | 115.50 | 123.50 | 137.80 | 89.40 |
| **FT3** | 4.41 | 3.95 | 4.81 | 4.20 | 4.29 | 4.04 | 4.81 | 4.81 | 3.13 | 3.95 | 3.98 | 4.50 | 3.63 |
| **FT4** | 17.38 | 19.72 | 18.46 | 13.58 | 11.25 | 13.72 | 13.55 | 12.66 | 14.49 | 11.57 | 14.13 | 15.47 | 13.47 |
| **TSH** | 3.073 | 5.521 | 5.034 | 4.430 | 9.473 | 8.111 | 13.304 | 8.175 | 3.760 | 7.621 | 9.837 | 10.746 | 6.020 |
| **TG** | 39.90 | 280.00 | 198.00 | 205.00 | 213.00 | 166.00 | 79.50 | 90.10 | 63.80 | 34.00 | 29.30 | 20.20 | 24.60 |
| **A-TG** | 17.90 | 19.60 | 20.20 | <15.00 | <15.00 | <15.00 | 24.90 | 24.70 | <15.00 | 21.90 | 16.00 | <15.00 | <15.00 |
| **A-TPO** | 40.00 | 38.10 | 34.30 | 44.90 | <28.00 | 43.90 | <28.00 | 40.50 | 48.10 | 35.00 | <28.00 | 29.40 | 44.60 |

**T3: triiodothyronine, T4: thyroxine, FT3: free triiodothyronine, FT4: free thyroxine, TSH: thyroid stimulating hormone, TG: thyroglobulin, A-TG: thyroglobulin antibodies, A-TPO: thyroid peroxidase antibody**
